# Eye and renal complication in pediatric patients with diabetes mellitus type 1

**DOI:** 10.1186/1687-9856-2015-S1-P23

**Published:** 2015-04-28

**Authors:** Nguyen Tran Ngoc Hieu, Bui PhuongThao, Vu Chi Dung, Nguyen Ngoc Khanh, Can Thi Bich Ngoc, Nguyen Thi Hoan, Nguyen Phu Dat, Truong Ngoc Duong

**Affiliations:** 1 103 Military Hospital, Hanoi, Vietnam; 2 Vietnam National Hospital of Pediatrics, Hanoi, Vietnam; 3 Hanoi Medical University, Hanoi, Vietnam

**Keywords:** Eye complication, renal complication, type 1 diabetes mellitus.

## 

Management and life expectancy for patients with diabetes mellitus type 1 (DM 1) is significantly improved. Long-term complication of the disease such as eye and renal complication have been paid much attention because it is an important cause of blindness and renal failure.

## Aims

We studied status of eye and renal complication and its relationship with HbA1C in 81 patients diagnosed of DM 1.

## Method

Observational, random collection.

## Results

Eye complication was seen in 23.5% of patients. Renal complication was seen in 22.2% of patients. 13.6% of patients had both kinds of complication. The longer duration of DM 1 the patients had, the more eye and renal complication was detected. In groups with eye and renal complication, HbA1c was significantly higher than that in groups without complication. Probability of having renal complication in a poor glucose control group was higher than that in a good glucose control group with OR ratio of 2.2. For eye complication, the figure was 3.7.

## Conclusion

The best thing to postpone eye and renal complication is glucose control.

